# Low Dimensional String-like Relaxation Underpins Superionic Conduction in Fluorites and Related Structures

**DOI:** 10.1038/srep44149

**Published:** 2017-03-27

**Authors:** Ajay Annamareddy, Jacob Eapen

**Affiliations:** 1 Department of Nuclear Engineering North Carolina State University, Raleigh, NC 27695, USA.

## Abstract

Among the superionic conductors that show a Faraday transition – the continuous increase in the ionic conductivity over a range of temperatures – the fluorite structures have enjoyed incisive examinations over the past four decades; yet the fundamental nature of superionicity has remained largely inconclusive. Departing from the traditional quasi-static defect framework, we provide weighty evidence for string-like dynamical structures that govern the fast ion conduction process in fluorites. We show that lower temperatures encourage the growth of longer but slowly relaxing strings and *vice-versa* – a direct manifestation of heterogeneous dynamics. Remarkably, the ionic conductivity is inversely correlated to the lifetime of the ions that participate in the strings and not explicitly to the ion population. Our analysis methodology, which resolves a long-standing disagreement on defect structures and the mechanism of ionic transport in *fcc* fluorite structures, is well-positioned to describe the dynamics of low dimensional conduction in a larger class of superionic conductors.

The existence of liquid-like conductivities in solid state ionics has been a constant source of puzzlement and a continual challenge for the experimentalists and theorists alike^1^,^2^,^3^. An impediment to uncovering the conduction mechanisms arises from the difficulty in characterizing the dynamic disorder for a subset of highly mobile ions that are constrained within a lattice framework^4^,^5^. The large amplitude anharmonic and asymmetric vibrations that are generally characteristic of the mobile ions further obstruct the construction of well-posed quasi-static defect models with a compelling association to the ionic conduction process.

Among the several factors that are known to enhance fast ion transport^6^ such as the smaller size of the ions and ionic polarizability, the availability and connectivity of sites for the mobile ions and a relatively low energetic barrier for migration^7^ appear to be essential for superionic conduction. Particularly, the body centered cubic lattice (*bcc*) framework has been noted to promote faster diffusion relative to close-packed structures such as face centered cubic lattices (*fcc*) and hexagonal cubic lattices (*hcp*)^8^. While a mobile ion can migrate between alternating octahedral and tetrahedral sites in an *fcc* lattice, the additional energetic barrier between them diminishes the ability to conduct ionic current^8^. The energetically equivalent tetrahedral sites in a *bcc* sub-lattice, on the other hand, is more accommodating for transporting charged ions; based on extensive first principles electronic structure simulations, a recent investigation arrives at the same conclusion for lithium-based superionic conductors^1^. While no single criterion envelopes the response of all superionic conductors, optimizing the substratal lattice arrangement is a promising direction for discovering new superionic conductors that are technologically relevant for energy storage and conversion^1^.

Drawing out the ion migration mechanisms within an *fcc* lattice poses peculiar challenges, which chiefly ensue from the paucity of excess lattice sites or from dynamic avoidance of certain unfavorable sites. In superionic *α*-CuI, for example, neutron scattering experiments and reverse Monte Carlo (RMC) modeling show that the mobile cations within an *fcc* anion lattice do not occupy the octahedral sites^9^; instead they chart out a distorted pathway between the tetrahedral sites along the edges of the octahedral cavity^7^. Thus the octahedral centers are density minimas while the tetrahedral sites correspond to density maximas^6^. The difficulty is more pronounced in fluorite structures such as UO_2_ and CaF_2_ that have a cation *fcc* structure with the mobile anions positioned at all available tetrahedral sites. In a more figurative representation, the anions are positioned at the vertices of a simple cube while the cations occupy the alternate cube centers. At low temperatures, the empty cube centers, which are the octahedral sites of the *fcc* cation lattice, accommodate interstitials forming Frenkel pairs^6^. At higher temperatures, when the structure crosses to the superionic state, the octahedral sites increasingly repel the interstitials forcing the anions to adopt conduits that are not dissimilar to those in *α*-CuI while hopping between native lattice sites. In fluorites, the ionic conductivity increases over a broad range of temperatures, which is termed as a Faraday transition^10^ or a Type II transition following the nomenclature of Boyce and Huberman^8^,^10^; in contrast, *α*-CuI portrays Type I transitioning to a highly conducting state following a thermodynamic phase change. Although the ions in Type I conductors typically undergo jump diffusion, the conduction process is often characterized as liquid-like because the time-averaged structure can be approximated by a random distribution of mobile ions over all available vacant sites^6^. With the octahedral sites exhibiting a somewhat mysterious reluctance in accommodating interstitials, such a random distribution model cannot be extended to the superionic state of binary fluorites.

To interpret the disorder in fluorites, defect cluster models populated by ions in relaxed positions, namely locations that are shifted away from geometrical vertices, have been proposed in the Eighties by Hutchings and coworkers^11^,^12^. These models are primarily quasi-static though with a latitude to accommodate dynamic evolution over short time periods that are of the order of picoseconds. While the dynamic cluster models, also labeled as dynamic Frenkel pairs (FPs), have been adopted over the past three decades as the preeminent theoretical scaffolding for extracting information on defects, they are largely silent on the actual ionic conduction process itself – an observation first made by Gillan^13^,^14^, who indicated that mobile species mostly hop between native sites in the superionic state. Thus the defects in the picture offered by Gillan are the anions that undergo dynamic displacements between lattice sites. The difference between these viewpoints is fundamental; in the defect cluster models, superionic conduction is presumably emerging from additional vacancies that are created by the cluster formation itself although a large number of high fidelity atomistic simulations, both classical and *ab initio*, have failed to establish their presence. In the Gillan picture, ion conduction is emanating from the ion hopping process itself albeit without a satisfactory exposition of the specifics of the jump behavior. The current analysis, which advances our recent work^15^,^16^ on fluorites that was motivated by the seminal insights elucidated by Gray-Weale and Madden^17^,^18^ on heterogeneous cooperative dynamics among the mobile anions, is directed at resolving this long-standing puzzle. Rather than appealing to the traditional defect hypothesis, we show, using atomistic simulations, that relaxation of low dimensional string-like structures governs the dramatic rise in ionic conductivity spanning several orders in magnitude. Our analysis thus supports the conceptualization rendered by Gillan and provides the missing mechanistic details and metrics of the ion hopping processes that underpin superionic conduction in fluorites and related structures.

## Results

We investigate two prototypical ionic conductors – UO_2_ and CaF_2_ – using molecular dynamics (MD) simulations. For achieving correlation times of 1 *ns* or more with several thousands of ions, classical MD with interatomic potentials is preferred over *ab-initio* simulations; predicted thermodynamic and transport properties show good agreement to experimental data^15^,^16^. In particular, the temperatures at the *λ*-transition – characterized by a peak in the specific heat and sometimes attributed to a second order phase transition – are determined to be 2560 *K* and 1455 *K* for UO_2_ and CaF_2_, respectively, and are in excellent accordance with the corresponding experimental data of 2610 *K* and 1430 *K*^16^.

### Evolution of Low Dimensional String-like Structures

Dynamic hopping from one anion site to another constitutes the most rudimentary ionic event in both UO_2_ and CaF_2_ at elevated temperatures – an observation entirely consistent with the neutron scattering data^11^,^12^. As the anions hop, often through peripatetic and meandering tracks, the simulations demonstrate that they engage in a string-like displacement process, where the ions follow each other in quasi one dimensional trajectories. We define strings as a set of ions constituted by pairs of mobile anions wherein one anion replaces another in an adjacent site over a certain time interval that is allowed to vary in the simulation^19^,^20^. Thus at the minimum, a string can involve a binary set of anions with one ion replacing the other. If *L*[*δt*] is the number of anions detected in a string over a time interval of *δt*, the mean string length Λ is computed as 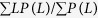, where *P(L*) is the probability of detecting a string across the same time interval^19^. We have varied *δt* from one tenth to several hundreds of picoseconds; the lowest value is chosen such that it is of the order of vibrational timescale of the anions. Since the mobile ions tend to move with ions of comparable mobility, it is desirable to group the ions of similar mobility to capture the (string-like) dynamics of the mobile ions efficiently. Ideally, a large number of mobile groups is desirable; however, with the limited number of ions in our investigation (4096 anions – see Methods section), a partitioning into 20 mobile groups, with ~205 anions (5% of the total) in each group, allows robust string identification with adequate statistics. In the sub-sections to follow, we shall first discuss the string dynamics of the most mobile group (top 5%); we then show that the string-like structures in different mobile groups are dynamically similar.

**Figure 1:**
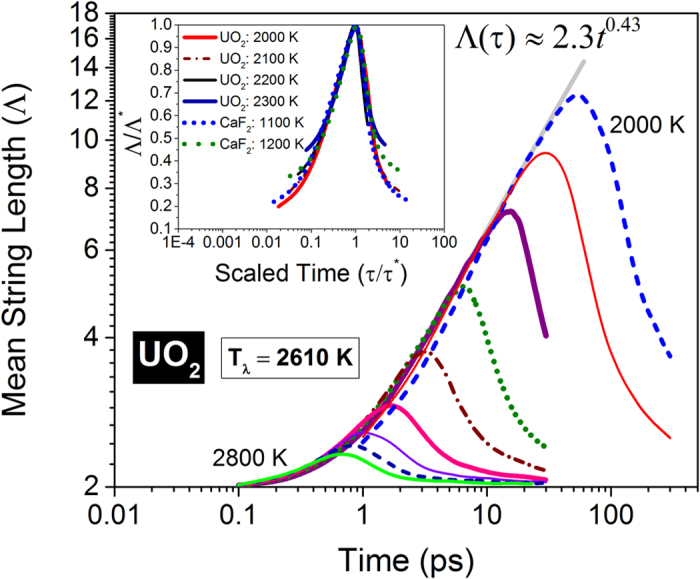
String Dynamics. Time evolution of string-like dynamic structures among the most mobile (top 5%) oxygen anions in UO_2_ for temperatures ranging from 2000 *K* to 2800 *K* in steps of 100 *K*. The mean string length Λ is measured by the number of ions constituting the string. A quantitatively similar behavior is exhibited by the fluorine ions in CaF_2_ (see Section I of the supplemental notes). The inset shows scaling similarity near the peak times.

In Fig. 1 we depict the temporal change in the mean length of the strings constituted by oxygen anions in UO_2_ for different temperatures ranging from 2000 *K* to 2800 *K* with the latter above *T*_*λ*_ = 2610 *K*^21^; a similar behavior is portrayed by the fluorine anions in CaF_2_ (see Section I of the supplemental notes). At all temperatures and for both conductors, the mean string length Λ increases first and then diminishes with a peak string length (Λ*) at a characteristic lifetime (τ*). Interestingly, at higher temperatures close to *T*_*λ*_, the string lifetime τ* is *O*(1) *ps*, which is in quantitative agreement with that of the disordered clusters evaluated from neutron scattering^12^. With decreasing temperature, the mean length embraces a power-law behavior for the growth regime, which interestingly is almost independent of the temperature; the growth rate of longer strings at low temperatures is thus almost identical to that of shorter strings at higher temperatures. String-like displacements are known to manifest in kinematically jammed states such as supercooled liquids^19^, colloids under shear^22^, polymer melts^23^ and granular materials^24^. Unlike in deeply supercooled states, there is a competition between dynamic facilitation^25^,^26^,^27^ – a characteristic of heterogeneous dynamics^28^ – and entropy which tends to destabilize the correlated dynamics in superionic conductors^15^,^16^,^17^. We will now discuss several unique features of the entropic-controlled jammed state in fluorite conductors and elucidate how the string-like structures govern the fast ion conduction process.

**Figure 2:**
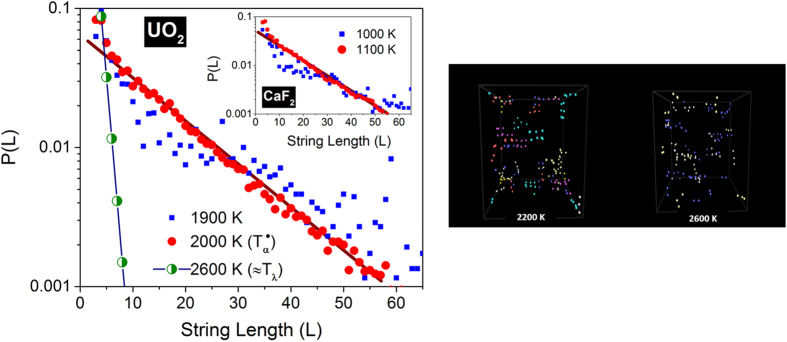
(Left) String probability distribution among top 5% mobile anions. The probability distribution *P(L*) for anions in UO_2_ at different temperatures evaluated at τ*. The temperature *T*_*α*_ corresponds to the onset of fast ion conduction in fluorites^15^,^16^ and connotes an order-disorder transition. *P(L*) for anions in CaF_2_ (inset) indicates that *T*_*α*_ is close to 1100 *K* from the simulations. Unlike in supercooled states and granular media, superionic conductors support longer strings involving several tens of ions. **(Right) Snapshots of string-like structures.** The spatial distribution of strings (evaluated at τ*) of different lengths at 2200 *K* (left) and 2600 *K* (right) in UO_2_. Different colors represent strings of different lengths. Longer strings that age slower are observed near *T*_*α*_ for both UO_2_ and CaF_2_, and *vice versa* near *T*_*λ*_.

At temperatures near *T*_*λ*_, the strings exhibit an exponential probability distribution; with decreasing temperature, the probability of finding longer strings increases rapidly as depicted in Fig. 2 (left panel). Below a characteristic temperature (*T*_*α*_), which is ~2000 *K* for UO_2_ (~1100 *K* for CaF_2_), the distribution strikingly forfeits its exponential character. The visible vestiges of the exponential form below *T*_*α*_ emanate from the less coherent and malformed string structures. Thus at temperatures above *T*_*α*_, for both UO_2_ and CaF_2_, several tens of ions form an ordered dynamical arrangement charting out quasi one dimensional dynamical tracks in three dimensional space – see snapshots in Fig. 2 (right panel); below *T*_*α*_, the anions progressively lose their ability for sustained string-like coherence. The well-defined lattice positions of the anions support very long string-like displacements unlike in supercooled liquids^20^ and granular media where the string lengths are much more restricted^24^. It is interesting to note that the distributions are similar near the transition temperature *T*_*α*_ for both CaF_2_ (see inset in left panel) and UO_2_. We have verified that the formation of string-like dynamical structures is independent of the type of interatomic potential that is used in the simulations. With a many-body potential using the embedded atom method (EAM) recently developed for actinides^29^, similar string-like formation and kinetics are observed in UO_2_ with a nearly identical temperature dependence; the results of a comparative study will be reported elsewhere.

**Figure 3:**
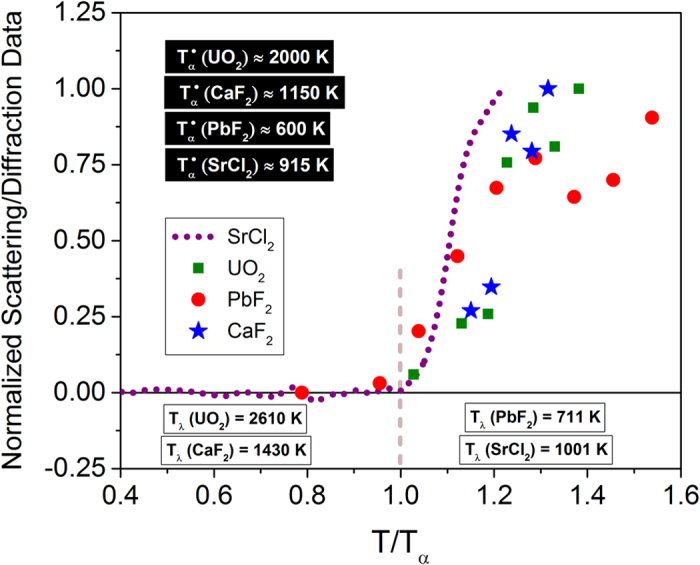
Normalized scattering/diffraction data showing an order-disorder transition at *T*_*α*_. The superscript (•) denotes inferred values from experimental data for UO_2_^12^, CaF_2_^11^, PbF_2_^31^ and SrCl_2_^5^. The current simulations show that anions which are thermally displaced at ~*T*_*α*_ engage in string-like displacements. Note that *T*_*λ*_ ≈ 1.25 *T*_*α*_ and 1.3 *T*_*α*_ for CaF_2_ and UO_2_, respectively.

Neutron scattering and diffraction experiments give the strongest and most definitive support for mobile anions that leave the native lattice sites before the *λ*-transition^4^,^5^,^11^,^30^,^31^. Figure 3, which shows a compilation of recent and historical scattering/diffraction data, strongly testifies to an order-disorder transition for several fluorite structures at a characteristic temperature *T*_*α*_; this crossover is also correlated to changes in ionic conductivity and other properties such as thermal expansion, specific heat, elastic constants and diffusivity although the variation is generally gradual and more subtle^15^,^16^ than that at λ-transition.

The neutron scattering/diffraction data in Fig. 3, normalized to the reported maximum value, include both integrated coherent diffuse scattering per anion (*X*^*D*^), and the fraction of disordered anions (*n*_*d*_) that is deduced by assuming a particular defect model^5^,^11^,^12^. The scattering measurements give information on ions that leave the native lattice sites, and hence, they provide a quantitative estimate on the dynamic disorder of the system. It is compelling to observe that there is a definitive onset of disorder, which is particularly evident from the recent investigation on SrCl_2_^5^, as well as for UO_2_^12^ and PbF_2_^31^, and the onset temperature *T*_*α*_ coincides with the formation of identifiable strings with low dimensionality. *Thus the current work elucidates the fate of the disordered ions in fluorite conductors, which has largely been left unsettled in the past*. While dynamic disorder has been characterized by short-lived clusters of defective anions^11^, they have not been successfully utilized for predicting the enhanced diffusion process. Indeed, the basic metrics such as ionic size, crystal structure and defect clusters have been considered to be insufficient for predicting the ionic conductivity even in one of the simplest superionic structures^4^. In the next sections, we will evaluate the characteristic lifetimes of the strings and demonstrate that the rapid rise in the ionic conductivity near *T*_*α*_ originates from the transport of strings with finite lifetimes.

### String lifetimes and heterogeneous dynamics

**Figure 4:**
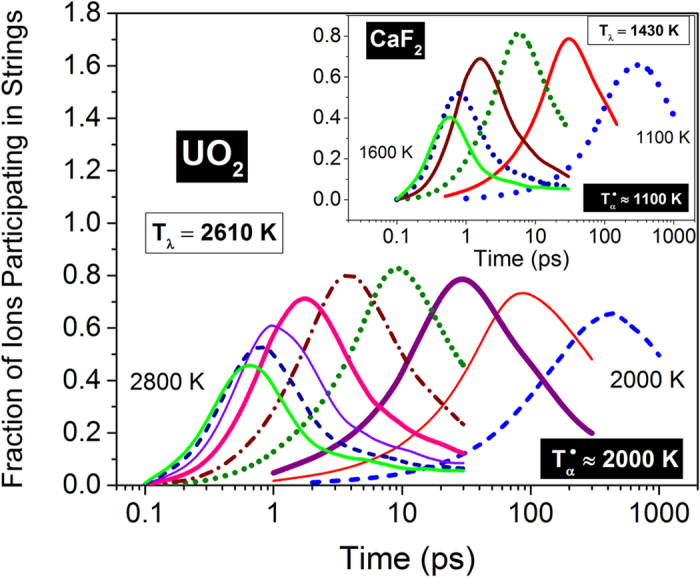
Participation of ions in strings among top 5% mobile anions. The fraction of anions that participate in strings in UO_2_ for temperatures ranging from 2000 *K* to 2800 *K* in steps of 100 *K*. The time at which most ions participate in strings (*τ*^*P*^) surprisingly does not correspond to the time at which the individual strings have most ions (τ*) at temperatures below *T*_*λ*_. The fluorine anions in CaF_2_ show a similar behavior for temperatures ranging from 1100 *K* to 1600 *K* in steps of 100 *K* as shown in the inset. The superscript (•) denotes inferred values from simulations.

In supercooled liquids, typically, the lifetime of the strings τ* is closely related to the representative lifetimes of heterogeneous dynamics^19^. In fluorite superionics, a rather surprising separation of timescales is noticed below *T*_*λ*_. Figure 4 delineates the time evolution of anion population in UO_2_ that participate in strings for different temperatures. The peak participation is almost 70% at *T*_*α*_ ≈ 2000 *K*, which grows to more than 80% at intermediate temperatures and then recede as the temperature approaches *T*_*λ*_, which is reminiscent of the waxing and waning of dynamical heterogeneity (DH) among anions in UO_2_^15^. A similar behavior is also observed for fluorine ions in CaF_2_ as shown in the inset. Rather surprisingly, the timescale associated with ion participation below *T*_*λ*_ is radically different from that of string growth, which is shown in Fig. 1. Thus we can identify two distinct timescales – the time at which the mean string length is maximum (τ*) and the time at which most ions participate in strings (*τ*^*P*^). Interestingly these lifetimes for supercooled liquids are of the same order of magnitude – see Section II of supplemental notes. While the former lifetime is intimately connected to the formation of individual strings, the latter is representative of ion participation among different strings.

**Figure 5:**
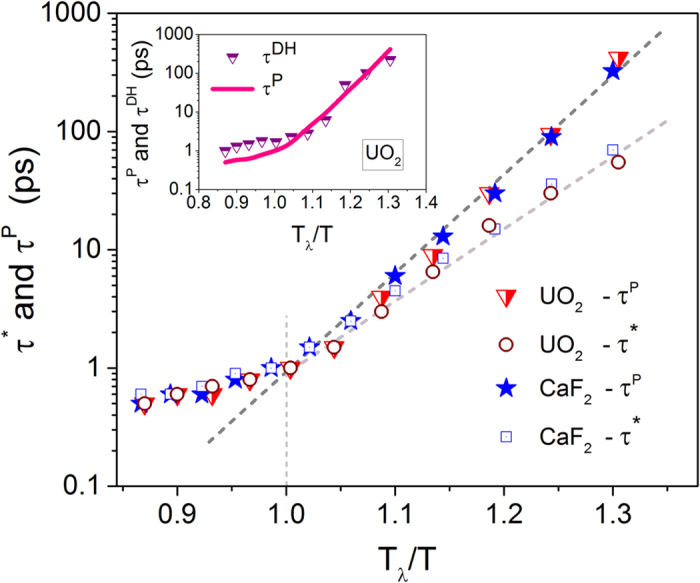
Peak lifetimes of strings among the top 5% mobile anions and timescale of dynamic heterogeneity (DH). Symbols τ*, *τ*^*P*^ and *τ*^*DH*^ correspond to the lifetime of strings, peak time at which most ions participate in strings, and the peak time when DH is maximum^15^. At *T*_*λ*_, the timescales converge and are *O*(1) *ps* for both UO_2_ and CaF_2_. Note that *T*_*λ*_ ≈ 1.25 *T*_*α*_ and 1.3 *T*_*α*_ for CaF_2_ and UO_2_, respectively.

We now delineate the lifetimes τ*and *τ*^*P*^ in Fig. 5 for different scaled temperatures. At temperatures *T*_*λ*_ and above, the two lifetimes are nearly identical to each other as in supercooled liquids; however, they diverge below *T*_*λ*_ although maintaining an Arrhenius variation. Interestingly, the corresponding timescales (*τ*^*P*^ or τ*) for UO_2_ and CaF_2_ are almost identical to each other when scaled to *T*_*λ*_, which hints at a universality in the string relaxation process among different fluorites. Consistent with neutron scattering experiments^11^,^12^, at temperatures *T*_*λ*_ and above, the lifetimes for both conductors are *O*(1) *ps* indicating a fast liquid-like relaxation mechanism, however, involving cooperative jumps between lattice sites. The unusual divergence between *τ*^*P*^ or τ* at temperatures below *T*_*λ*_ reveals that the dynamics at the level of individual strings is not controlling the relaxation of the system, which determines the properties such as ionic conductivity. In our earlier work^15^ we have determined that Type II superionic conductors exhibit dynamic heterogeneity (DH), which waxes and wanes with increasing temperature. The DH makes its first appearance at *T*_*α*_, exhibits a peak and then diminishes as the temperature approaches *T*_*λ*_. The rise and fall of heterogeneous dynamics is also confirmed through a similar behavior in dynamic facilitation (DF) – a closely related metric which forecasts that mobile particles foster neighboring particles to become mobile thereby allowing mobility to propagate continuously in a spatially correlated manner leading to large scale DH^25^,^26^,^27^. While the intensity of DH, evaluated through a space-time correlation of propensity^15^, shows a non-monotonic variation with temperature, the peak time at which DH has the highest magnitude (*τ*^*DH*^) decreases monotonically as shown in the inset of Fig. 5.

**Figure 6:**
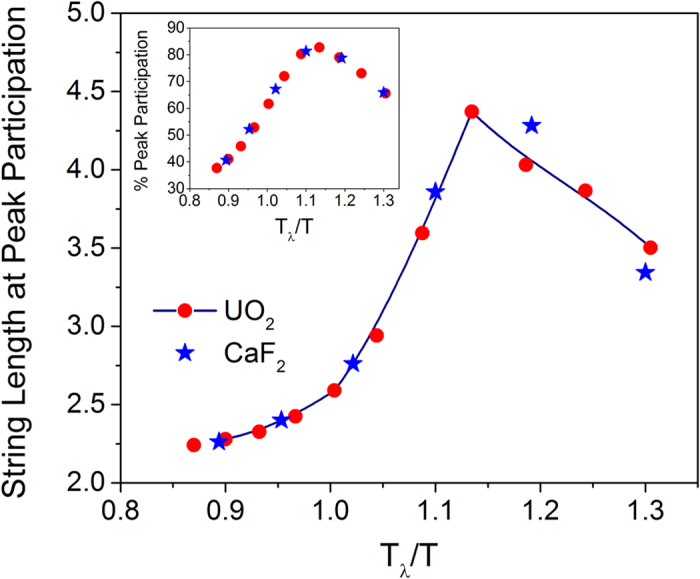
Variation of mean string length at *τ*^*P*^ with temperature among top 5% mobile anions. The inset shows the peak participation (%) at different temperatures. The longest string at *τ*^*P*^ (~4.3) occurs at a temperature corresponding to the peak participation of ions (~82%) indicating a spatial correlation among strings. As the temperature approaches *T*_*λ*_, the string length converges to ~2, which indicates simultaneous hopping of two anions.

It is remarkable to note that the peak time for most ions to participate in strings (*τ*^*P*^) shows a strong correlation to *τ*^*DH*^ thereby establishing a vital but hitherto hidden association between the relaxation process induced by string-like structures and heterogeneous dynamics in fluorite superionic conductors. Interestingly, the lifetime for strings has been shown recently to correlate to diffusional processes in supercooled liquids, while a similar correlation has been observed between the structural relaxation time and the lifetimes of clusters comprising of immobile particles^32^. The peak lifetime of the strings τ* being smaller than the peak participation time *τ*^*P*^ brings out another interesting variation in the string kinetics. Near *T*_*α*_, the lifetime τ* is almost an order of magnitude smaller than *τ*^*P*^, which indicates that longer strings have lost their coherency and have dissipated to become shorter strings at *τ*^*P*^. Thus the mean string length at *τ*^*P*^ shows a non-monotonic variation with temperature as depicted in Fig. 6.

The logical picture that ensues from the preceding analysis is as follows: At temperatures close to *T*_*α*_, well-defined string-like structures are formed, which accommodate a large proportion of participating ions. These low dimensional dynamic structures, although spanning the three dimensional space, are in essence the dynamic disorder identified in scattering experiments of Type II ionic conductors. The metric that is paramount to characterizing disorder is not the spatial locations which the strings assume but it is through the string lifetimes. Because the lifetime of individual strings is considerably shorter than that of peak participation lifetime, the peak string length, which involves no more than five participating ions for both UO_2_ and CaF_2_, occurs at an intermediate temperature between *T*_*α*_ and *T*_*λ*_ – a behavior which is remarkably similar to the waxing and waning of DH reported in our earlier work^15^,^16^. Since the DH lifetime (*τ*^*DH*^) is close to the peak participation lifetime (*τ*^*P*^) as shown in the inset of Fig. 5, we thus arrive at the compelling conclusion that the collective ionic participation among many string-like structures promotes heterogeneous dynamics in the arrested state of fluorite conductors.

### Diffusivity and Ionic Conduction from String Dynamics

**Figure 7:**
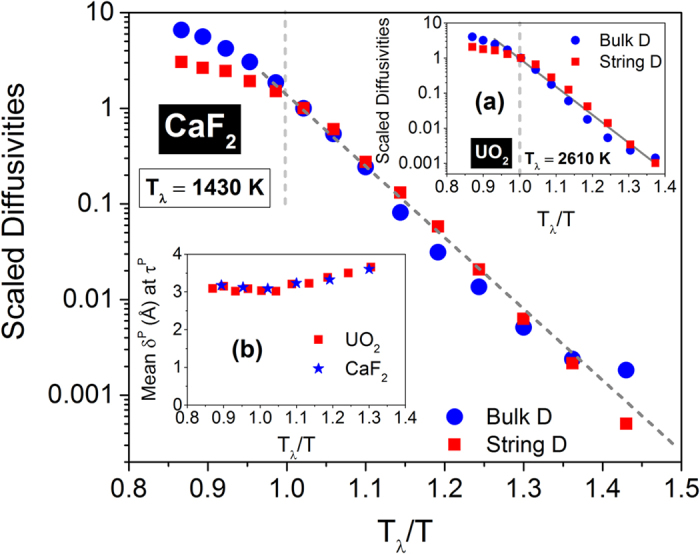
Comparison of scaled diffusivities for CaF_2_ and UO_2_. The string diffusivity is evaluated as (*δ*^*P*^)^2^/*τ*^*P*^. The diffusivities are scaled to their respective magnitudes at *T*_*λ*_. The mean string displacements (*δ*^*P*^) at peak string participation lifetime *τ*^*P*^ are depicted for UO_2_ and CaF_2_ in inset (***b***).

We will now endeavor to associate the self-diffusivity of the system evaluated from the mean square displacement of all anions (Δ*r*^2^) to the mobility of the strings. The strings that are formed in the superionic state are transient and spatially varying with step-like displacements. Since the string-like structures are dynamically formed and annihilated beyond a certain time period, the regular statistical-mechanical expression for diffusivity as the infinite-time limit of the slope of the mean square displacement becomes inadequate. We have, therefore, assumed that the strings govern the diffusion process within a certain characteristic timescale, and have calculated an effective string diffusivity based on the mean displacement of the strings (*δ*^*P*^) at the peak participation lifetime (*τ*^*P*^), which also scales with the DH timescale. The effective string diffusivity is then calculated as the time average of (*δ*^*P*^)^2^/*τ*^*P*^ analogous to the slope of the mean square displacement of the ions. In Fig. 7, we compare the mean self-diffusivity of all the anions evaluated as lim *t* → ∞ (Δ*r*^2^/6*t*) with the mean string diffusivity scaled to the corresponding values at *T*_*λ*_.

The striking observation from Fig. 7 is that the effective string diffusivity of both CaF_2_ and UO_2_ (shown in inset (*a*)) increases almost at the same rate with temperature as the self-diffusivity, and nearly are identical to each other. Both show an Arrhenius variation with temperature (up to *T*_*λ*_) and are highly correlated to each other for more than three orders of magnitude. Inset (*b*) shows that the mean string displacements at *τ*^*P*^ for both UO_2_ and CaF_2_, which are only slightly more than one anion lattice constant, are virtually identical and differ little across the high temperature superionic state. Thus the string diffusivity is largely determined by the peak participation lifetime *τ*^*P*^, and only weakly on *δ*^*P*^. It is also instructive to note that the string lifetime τ* fails to predict the change in the bulk self-diffusivity; in Section III of the supplemental notes, we depict the comparison of the scaled string diffusivities with τ* and *τ*^*P*^.

The closely knit correlation between the effective string diffusivity and bulk self-diffusion underscores the significance of the low dimensional string-like dynamic structures. Not only do the strings account for the disorder in the superionic state, which is emphatically established by the scattering experiments, their kinetics can also quantitatively predict the order of magnitude change in the system self-diffusivity; such a mechanistic underpinning has been lacking in the past.

The final and the most pertinent observation we make is the correlation between the rapid increase in the ionic conductivity before it approaches typical liquid state values, and the dynamics of the string-like structures. If the current hypothesis is correct, the ionic conductivity should emanate from the transport of the strings itself. First, we note that the ionic conductivity can be computed exactly from the multicomponent linear response or Green-Kubo formalism^33^,^34^ as the time integral of the ionic current autocorrelation function. For weakly polarizable ionic conductors such as CaF_2_, the Nernst-Einstein (NE) relationship, which expresses ionic conductivity in terms of the self-diffusivity of the ions, generally shows reasonable agreement to the experimental ionic conductivity^35^. We will therefore adopt the NE model for evaluating the ionic conductivity; it is given by^36^


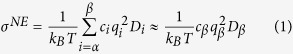


where *c*_*i*_, *q*_*i*_ and *D*_*i*_ are the concentration or number density, charge and self-diffusivity of the *i*^*th*^ species, respectively; *α* and *β* represent the cations and anions. In contrast to the anions in fluorites, the cations are mostly immobile and thus the ionic conductivity can be evaluated on the basis of the anion diffusivity alone.

**Figure 8:**
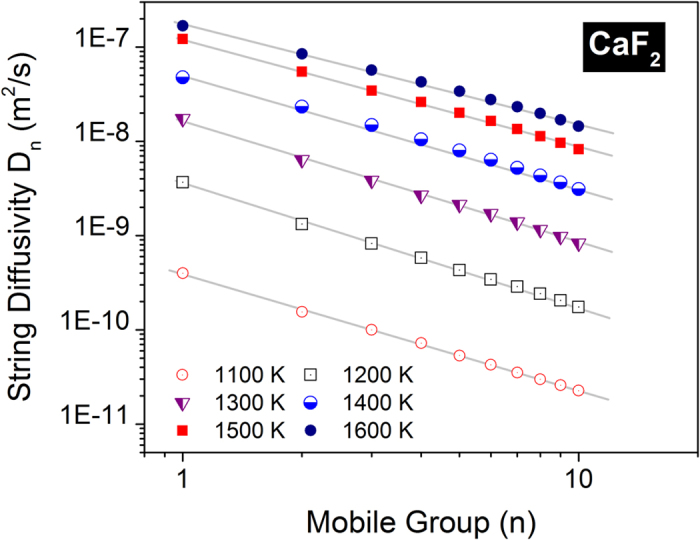
String diffusivity for different mobile groups covering 50% of the fluorine anions in CaF_2_. The string diffusivity is well-reproduced by a power-law of the form *D*_*n*_(*T*) = *a(T)n*^*b(T*)^ for all the temperatures.

The next step is to evaluate an effective diffusivity of the anions from the metrics that describe the string dynamics. Since the string diffusivity portrayed in Fig. 7 is assessed only from the top 5% of the mobile anions, a realistic appraisal will entail similar evaluations for the remaining anions. The anions are therefore portioned into groups of 5% and an effective string diffusivity for each group is then calculated using the same methodology used for the top 5%. In Fig. 8 we delineate the string diffusivity for the first ten mobile groups that covers the most mobile ions (50% of the anions) in CaF_2_. Interestingly, the string diffusivity shows a power-law scaling with different mobile groups which indicates an analogous increase in the peak participation lifetime (*τ*^*P*^) for the slower mobile groups; expectedly, the mean string displacement at *τ*^*P*^ does not show a significant variation. We have fitted a power-law variation of the form *D*_*n*_(*T*) = *a(T)n*^*b(T*)^ where *a* and *b* are two temperature-dependent constants for each mobile group. We also note that the string formation among the least mobile groups becomes increasingly lethargic and incoherent with no strings detected in the two least mobile groups. As evident from Fig. 8, the power law scaling is very robust and it allows the computation of a conservative estimate of the string diffusivities of the least mobile groups through extrapolation where string formation is stalling and statistically difficult to detect (no strings were detected in the last two groups). We have further verified that the string diffusivity from the power law extrapolation for the least mobile groups remains close to the value obtained directly from the simulations wherever strings can be identified. In Section IV of the supplemental notes, the analogous power-law scaling for string diffusivity among different mobile groups in UO_2_ is portrayed.

Having assessed the individual diffusivities of each string group, the final step involves appraising an effective diffusivity for the anions. Since ion mobility scales as diffusivity in ionic conductors, we adopt the well-known Matthiessen rule originally proposed for electron mobility – it states that the effective ionic resistivity of the system is simply a sum of the ionic resistivities of each group. Thus the effective or coarse-grained diffusivity is lower than the lowest diffusivity of any mobile group. In the past, Matthiessen rule has been successfully applied to electron and ion transport, and it is also an integral step in the calculation of thermal conductivity using Boltzmann transport equation^37^. While the original formulation is based on scattering lifetimes, the current application is based on string lifetimes of different mobile groups, which are independent of each other in the computational algorithm. The effective diffusivity 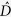 of the system, based on Matthiessen rule, can be expressed as


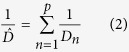


where *p* is the total number of mobile groups among the anions. Our criterion for choosing the ion population, in steps of 5% (constituting a total of 20 mobile groups), is empirically based on extracting robust string dynamics and is consistent with earlier investigations on supercooled liquids^19^,^20^. The power law gives an opportunity to further refine the effective diffusivity by considering a continuum of mobile groups; this approach also alleviates the drawback of arbitrarily assigning discrete mobile groups. Using the continuum approximation, the discrete relationship in Eqn. (2) can be recast as


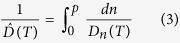


The Nernst–Einstein ionic conductivity based on effective string diffusivity and the power law can now be evaluated analytically among *p* groups of anions as


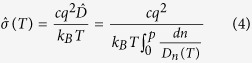


where *c* and *q* represent the anion concentration (number density) and ionic charge, respectively. The prediction of the continuum approach is only ~10% lower than that from the discrete summation in Eqn. (2), which is indicative of the insensitivity of any coupling that may exist between group diffusivities. We note here that our primary emphasis is on estimating the order-of-magnitude of the contribution from the strings to the overall (bulk) diffusivity and ionic conductivity.

**Figure 9:**
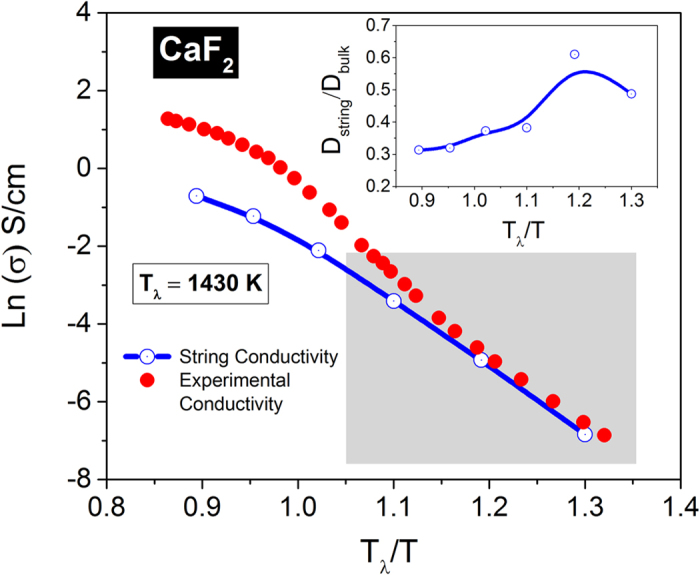
Nernst-Einstein (NE) ionic conductivity and diffusivity for CaF_2_. The string conductivity is evaluated from different mobile anion groups. The shaded portion depicts the temperature range where the contribution from the strings to the diffusion process is expected to be the highest (also see Fig. 6). The ratio of the string diffusivity to that of the bulk is shown in the inset. The estimated *T*_*α*_ from simulations is ~1100 K.

In Fig. 9, we compare the ionic conductivity calculated from the modified NE relationship against the experimental data^38^ for CaF_2_. The most striking observation is the close correspondence between the NE conductivity based on string diffusivity at low temperatures where strings start to form (near *T*_*α*_). As shown in the inset, the bulk diffusivity of the anions can principally be accounted by the string kinetics (~50–60%) near *T*_*α*_. With increasing temperature, the string contribution diminishes to ~30%, which is consistent with the reduction in dynamic heterogeneity near *T*_*λ*_^16^. It is quite remarkable that one single metric – the peak participation lifetime (*τ*^*P*^) – is largely capable of predicting the bulk diffusivity and ionic conductivity at temperatures where string-like dynamical structures are most likely to form and proliferate. This behavior is again consistent with the observation in Fig. 4 that fewer ions participate in strings near *T*_λ_ and above. Thus the uncorrelated anionic jumps are primarily responsible for diffusion and ionic conductivity near *T*_*λ*_ while correlated string-like structures underpin the ion transport near *T*_*α*_ (~1100 K from the simulations).

## Discussion

The discovery of string-like dynamical structures – a manifestation of heterogeneous dynamics^28^ – facilitates a mechanistic description of the dominant process that is associated with the transitioning to a superionic state. We have shown that strings proliferate rapidly near a characteristic temperature *T*_*α*_ that marks the onset of superionic conduction in fluorite structures^15^; at temperatures well below *T*_*α*_ strings disappear and do not contribute to the overall diffusion process. We thus resolve a long-standing puzzle on the fate of the anions that are known to leave the lattice sites from scattering experiments and affirm the ion-hopping mechanism proposed in the seminal work of Gillan^13^,^14^. While the anions at sufficiently high temperatures can take circuitous and peripatetic routes in a lattice circumvented by the cations, there appears to be no ambiguity on the most predominant anion pathway, which is from one native site to another with a residence time that is determined by the thermodynamic state. A significant accomplishment of our analysis is in showing that the peak participation lifetime of the ions (*τ*^*P*^) controls the effective diffusivity and ionic conductivity of the system; the same lifetime is also correlated to the peak lifetime of dynamic heterogeneity (*τ*^*DH*^). Complex defect clusters that have been proposed in the past appear to be redundant and the kinetics of the string-like structures are mostly sufficient to predict the diffusivity in fluorites. It is heartening to note that the string kinetics alone is able to offer a quantitative prediction that agrees closely with the experimental ionic conductivity at temperatures close to *T*_*α*_ in CaF_2_. The subtleties of multiple timescales associated with string formation and ion participation, which appear to be unique to superionic conductors, will be described in more detail in a later publication.

We will now contemplate on the potential impact of our discovery for a wider class of superionic materials. The preeminence of *bcc* sub-lattices in promoting superionic conduction is now well-established^1^ – this viewpoint is supported by the presence of energetically favorable and similar tetrahedral sites. Our findings indicate that superionic conduction process can be enabled through a low dimensional transport process involving string-like structures even when such empty sites have a relatively high energetic barrier. This picture is fundamentally different from the prevailing notion of examining the static defect positions and associated energetics – *in our viewpoint, fast ion conduction can be facilitated if the system is driven to a jammed state that will engender percolating low-dimensional structures*. Jamming can occur when accessible sites are energetically unfavorable such as the octahedral sites in fluorites or when mobile ions exceed the number of favorable sites.

The evidence from the current work appears to be persuasive to consider a broader applicability; evidently, our hypothesis needs to be tested with both experiments and simulations before arriving at a definitive conclusion. If the conjecture is proven correct, there appears to be a thermodynamic window for sustaining the jammed state even in conductors with close-packed sub-lattices. With increasing temperature, the crystalline order will attempt to stabilize the jammed state while the entropic forces will tend to destabilize it. From a practical viewpoint, this dynamic mechanism can potentially be examined for developing more efficient or specialized superionic conductors by manipulating the sub-lattice structure, ionic composition or thermodynamic/stress states. Given the characteristic string-like transport in severely constrained systems such as granular media and glassy states, it is plausible that the predominant one-dimensional ionic transport observed in lithium superionic conductors such as Li_x_FePO_4_^39^,^40^, Li_10_GeP_2_S_12_^41^,^42^,^43^ and LiMgSO_4_F^44^ is a consequence, at least partially, of a dynamic jammed state. The formalism developed in our work appears to be well-positioned for drawing out the dynamical origin of conductivity in a larger class of superionic conductors.

## Methods

### Atomistic Simulations

The kinetics of string-like structures in UO_2_ and CaF_2_ are evaluated through classical atomistic simulations using rigid-ion two-body interionic potentials that have been benchmarked to a wide range of properties^13^,^45^. CaF_2_ is modeled using a potential of the form^13^:


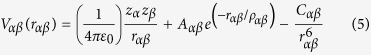


where the indices *α* and *β* label the ionic species, *z* is the charge, *r*_*αβ*_ denotes distance between the ions, and *A* and *C* are two constants. The first term in Eq. (5) represents the Coulombic interaction, while the second and third terms represent the repulsive potential due to the overlap of electron clouds and the attractive van der Waals potential, respectively; the effect of polarization is accommodated by the dispersion term^35^. For UO_2_, an additional Morse term (*D*) is included to describe the covalent bonding as shown below^45^. The numerical values of the interionic parameters for both conductors are given in our earlier work^16^.


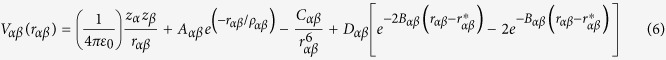


The simulations are performed with periodic boundary conditions in a cubic system with 6144 ions. The Newton equations of motion are integrated using the Velocity-Verlet algorithm with a time step of 1 *fs*. The system is initially equilibrated in an NPT ensemble for 25 *ps* at zero pressure; the properties are then evaluated in an NVE ensemble and averaged over 10 independent runs each with different initial conditions. The standard deviation in the dynamic properties from different independent runs is less than the size of the symbols used in the figures. The Coulombic interactions are simulated using the Wolf summation method^46^, which we have benchmarked to the standard Ewald sum method. It is observed that the total energy is conserved to five significant digits over hundreds of picoseconds (see Sec. V of the supplemental notes). We have verified that the potentials reproduce the characteristic transition temperatures as listed in Table 1 in addition to other thermodynamic and transport properties such as specific heat and self-diffusivity^15^,^16^.

In a recent work^47^, we have further shown that the interionic potential used for UO_2_ is able to predict the quasi-static disorder observed using neutron scattering and diffraction experiments at temperatures above 2000 *K*. We have further established recently that *T*_*α*_ corresponds to a well-defined entropic crossover in fluorite superionic conductors^48^.

## Additional Information

**How to cite this article:** Annamareddy, A. and Eapen, J. Low Dimensional String-like Relaxation Underpins Superionic Conduction in Fluorites and Related Structures. *Sci. Rep.* **7**, 44149; doi: 10.1038/srep44149 (2017).

**Publisher's note:** Springer Nature remains neutral with regard to jurisdictional claims in published maps and institutional affiliations.

## Supplementary Material

Supplementary Information

## Figures and Tables

**Table 1:** Comparison of transition temperatures –simulations and experiments.

	UO_2_	CaF_2_
*T*_m_ (*K*)	3125 (3120)	1600 (1633)
*T*_λ_ (*K*)	2560 (2610)	1455 (1430)
*T*_α_ (*K*)	2000 (2000)	1100 (1150)

*T*_*λ*_ and *T*_*m*_ correspond to the λ-transition temperature and the melting point, respectively^16^; the numbers in the parenthesis denote experimental values. *T*_*α*_ denotes the order-disorder transition temperature that coincides with the crossover to the superionic state (onset of superionicity).
