# Supplementary material for: Low Dimensional String-like Relaxation Underpins Superionic Conduction in Fluorites and Related Structures
Source: Sci Rep. 2017 Mar 27;7:44149. doi: 10.1038/srep44149 (PMC5366808; doi:10.1038/srep44149)
Supplement: Supplementary Information [file srep44149-s1.pdf]

Supplemental Notes

Low Dimensional String-like Relaxation Underpins Superionic Conduction in Fluorites and Related Structures

Ajay Annamareddy and Jacob Eapen*
Department of Nuclear Engineering
North Carolina State University, Raleigh, NC 27695
*jacob.eapen@ncsu.edu

Section I: Evolution of string length in CaF_2 with top 5% mobile ions

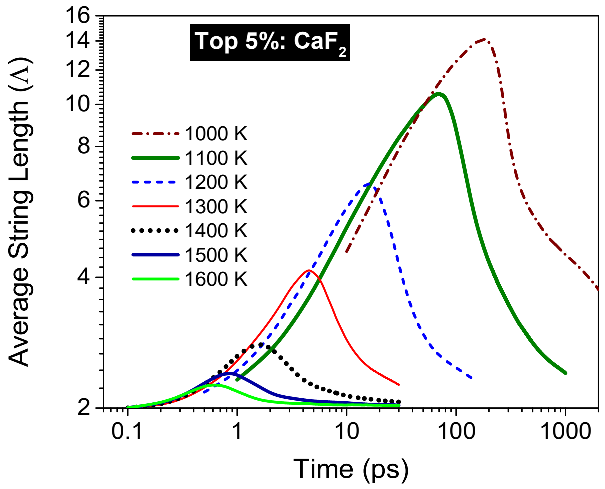

Figure S1 | Temporal evolution of mean string length in CaF_2.

Section II: String lifetimes in typical supercooled liquids

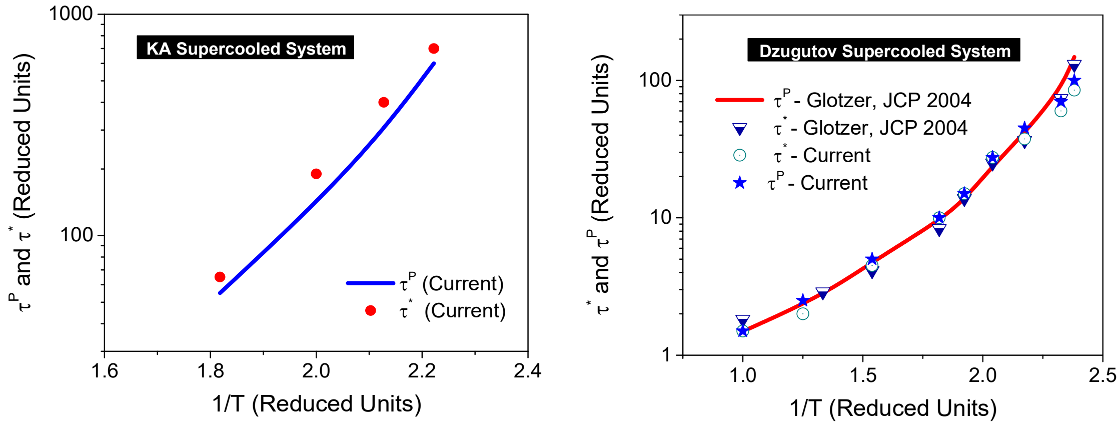

Figure S2 | String lifetimes in typical supercooled liquids.

Figures S2 shows the string lifetimes for the Kob-Anderson (KA) supercooled liquid[1,2] (left) and Dzugutov supercooled liquid[3] (right). The symbols τ^* and τ^P correspond to the peak lifetime of strings, and peak time at which

most ions participate in strings, respectively. As evident, these timescales are of the same order of magnitude for typical supercooled liquids.

Section III: Scaled string diffusivities with τ^* and τ^P in CaF_2

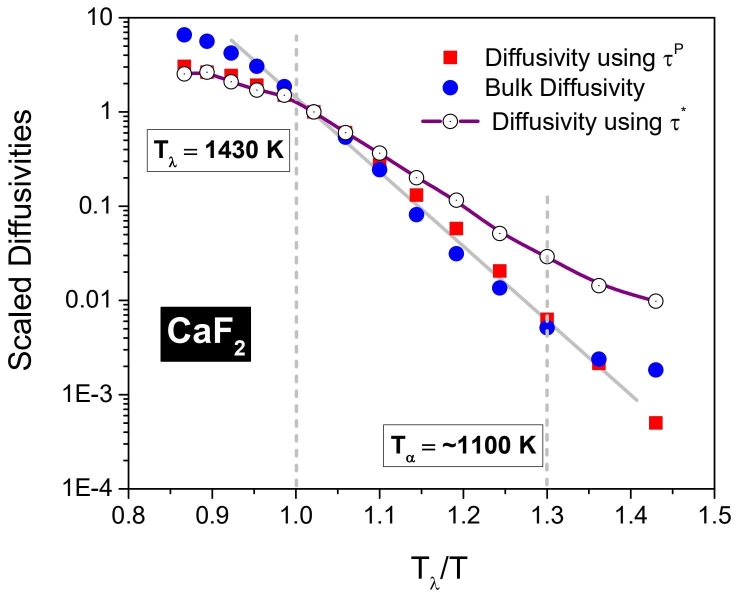

Figure S3 | Scaled string diffusivities using τ^* and τ^P in CaF_2; very similar behavior is also observed in UO_2.

Section IV: Power-law behavior of string diffusivity among different mobile anion groups in UO_2

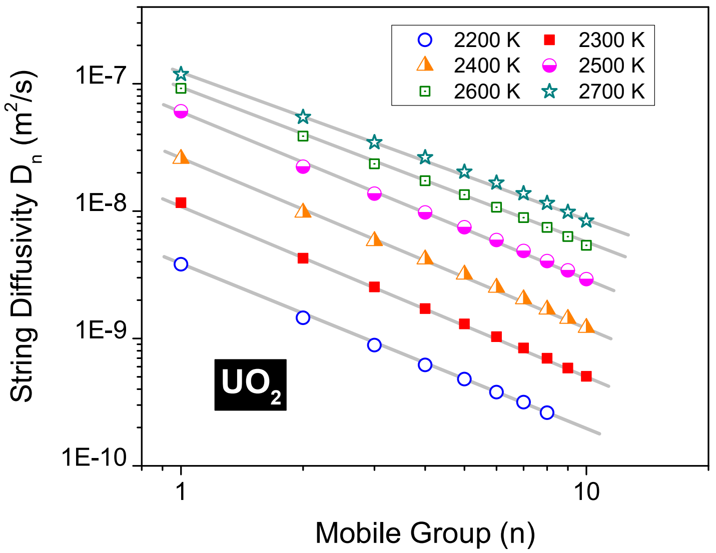

Figure S4 | String diffusivities for the most mobile groups in UO_2.

In Figure S4, we show the variation of string diffusivity of the most mobile groups among the oxygen anions in UO_2. The diffusivities are well described by a power-law similar to that for CaF_2 (see Figure 8 in the main text).

Section V: Energy conservation

System equilibration is verified through energy conservation and maintaining a constant (zero) pressure. Unlike supercooled liquids, the superionic systems are in thermodynamic equilibrium; therefore, a short equilibration time of 25 *ps* is generally sufficient for maintaining the system energy over hundreds of picoseconds.

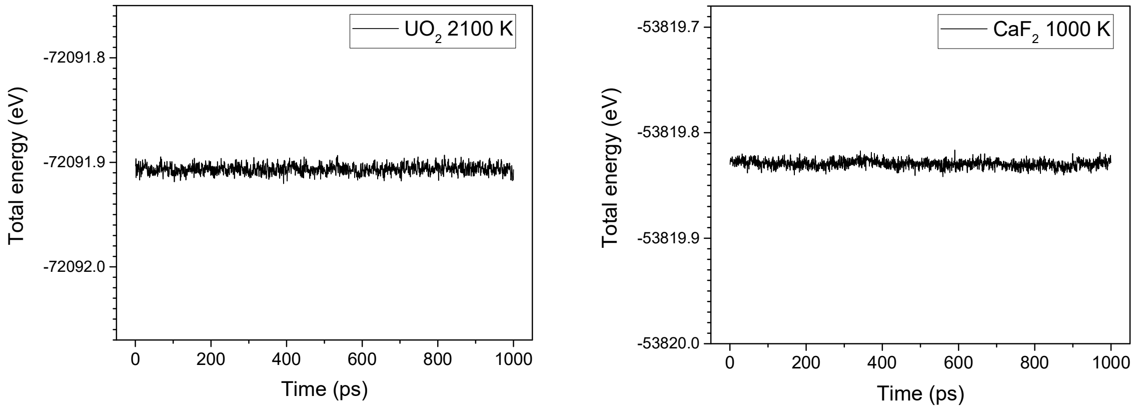

Figure S5 | Energy conservation in UO_2 (left) and CaF_2 (right) at temperatures near T_α. Total energy is conserved to five significant digits over several hundred picoseconds in all the simulations.

References

1 Kob, W. & Andersen, H. C. Testing mode-coupling theory for a supercooled binary Lennard-Jones mixture I: The van Hove correlation function. *Phys. Rev. E* **51**, 4626 (1995).

2 Kob, W. & Andersen, H. C. Testing mode-coupling theory for a supercooled binary Lennard-Jones mixture. II. Intermediate scattering function and dynamic susceptibility. *Phys. Rev. E* **52**, 4134 (1995).

3 Gebremichael, Y., Vogel, M. & Glotzer, S. C. Particle dynamics and the development of string-like motion in a simulated monoatomic supercooled liquid. *J. Chem. Phys.* **120**, 4415-4427 (2004).
